# Functionalized nanoparticles based on β-chitosan and styrene copolymer derivative for donepezil delivery

**DOI:** 10.1007/s13205-025-04425-z

**Published:** 2025-08-25

**Authors:** Fahima M. Helaly, Ahmed A. F. Soliman, Eman AboBakr Ali

**Affiliations:** 1 Polymers and Pigments Department, Chemical Industries Research Institute, National Research Centre (Scopus Affiliation ID 60014618), Dokki, Giza, 12622 Egypt; 2 Department of Pharmacognosy, Pharmaceutical and Drug Industries Institute, National Research Centre (Scopus Affiliation ID 60014618), Dokki, Giza, 12622 Egypt

**Keywords:** β-Chitosan-based polyelectrolyte complex, Sulfonated styrene–maleic anhydride, Nanogel, Donepezil hydrochloride, Acetyl cholinesterase inhibitory

## Abstract

Developing a drug delivery strategy that can cross the blood–brain barrier is crucial to effective neurological treatment. In this work, a new strategy was introduced for efficient drug delivery of Donepezil based on the preparation of polyelectrolyte complexes (PEC) nanogel from β-chitosan (CS) and the prepared sulfonated styrene–maleic anhydride (S-SMA). First, low-molecular-weight SMA was prepared. Then, sulphonation of SMA was carried out. Three PEC nanoparticles were prepared by mixing three different ratios of S-SMA with β-chitosan. The structure and characteristics of the nanoparticles were thoroughly investigated. Varying S-SMA content (donated CS-S1, CS-S2, and CS-S3) for fixed a β-CS content, the surface charge and average size of the nanoparticles were tunable*.* Donepezil hydrochloride (DH) was encapsulated successfully in the nanoparticles CS-S3 and donated as CS-S3-DH. Additionally, the transmission electron microscopy (TEM) images revealed that almost 50% of the nanoparticles particles had diameters of 27 ± 0.1 and 111 ± 0.4 nm for CS-S3 and CS-S3-DH, respectively. The in vitro drug release study indicates a sustained release of DH for 72 h. In addition, the in vitro acetylcholinesterase (AChE) inhibitory was investigated. The result showed that AChE inhibitory percentages were 16.5 and 63.9% for CS-S3 and CS-S3-DH, respectively.

## Introduction

Over decade, drug delivery system (DDS) based on polymeric materials has gained great attention. As this approach leads to design smart medications, that decreases the side effect of numerous drugs (Tong et al. 2020). Whether derived from natural or synthetic sources, polymers possess excellent flexibility to achieve various properties, ultimately resulting in the desired controlled release profile (Jagtap et al. 2021) (Fundueanu et al. 2021).

One promising class of polymers is polyelectrolytes which possess repeating negative or positive charge units in ionizing solvents nearby neutral pH (Lankalapalli and Kolapalli 2009; Jagtap et al. 2021). The unique properties of polyelectrolytes enable targeted and regulated delivery. Compared to uncharged polymers, polyelectrolytes possess unique characteristics including exceptional water solubility and swelling (Buriuli and Verma 2017a; Horkay 2021). In addition, polyelectrolytes have the ability to interact with neutral particles, small ions and oppositely charged surfaces or macromolecules (WanaSingha et al. 2021).

Polyelectrolytes have three categories depending on their origin: natural, synthetic, and chemically modified biopolymers. Among all, biopolymers have a high candidacy in the pharmaceutical sectors from a commercial point of view (Dakhara and Anajwala 2010) due to their availability from natural resources and ease of processing.

Chitosan (CS) is a cationic polyelectrolyte, chemically modified biopolymer, that has a significant impact in many applications (Ali et al. 2020; Hamed et al. 2022a). Chitosan has great potential in medical applications considering its inherent features from its origin, chitin (Nada et al. 2020; Ali et al. 2023; Kazemi Asl et al. 2023). Owing to CS’s biocompatibility, degradability, and low cytotoxicity, this biopolymer has been considered a safe carriers in drug delivery system (Nada et al. 2019). Moreover, CS has the ability to form polyelectrolyte complexes (PEC) with many different anionic polymers (Dacoba et al. 2019).

Many chitosan-based PEC were prepared with various polyanions for various applications (Ali et al. 2020; Wu et al. 2020). In biomedical field, semi-IPNs (PECs) were prepared by blending CS derivatives with carboxymethyl chitosan (CMC) and graphene quantum dots (GQDs) for wound healing (Hamed et al. 2022b). Another research tested alginate/chitosan PEC scaffolds with controlled porosity for tissue repair and regeneration (Bushkalova et al. 2019). In the same context, fibrous scaffolds were prepared for heart tissue engineering from the combination of CS and polylactic acid (PLA) (Kazemi Asl et al. 2023). Many natural or synthetic polymers have been implemented for CS-PEC preparation (Buriuli and Verma 2017b; Jagtap et al. 2021). However, the usage of copolymers to create CS-PEC has received less attention. Copolymers offer more chemical flexibility and functional groups, leading to improved applications.

Recently, styrene–maleic anhydride (SMA) copolymers have regained interest as a promising biocompatible polymer in the biomedical field (Tamayo et al. 2022). Utilizing SMA paved the way for producing new functional polymers through additional modifications of the anhydride or styrene groups. Earlier, In 1991, butyl monoester SMA was employed in cancer chemotherapy as conjugate with neocarzinostatin (Maeda 1991). In another research, the SMA copolymer was used to deliver Cuproferrogel, a female fertility control molecule combined with dimethyl sulphoxide (DMSO). This combination has demonstrated both long-term contraceptive efficacy and safety (Jha et al. 2010). Recently, new bioconjugates using post-polymerization modification of SMA with azidopropyl amine to produce new protein–polymer bioconjugate by copper-free click chemistry reaction (Saxer et al. 2022). Other researchers developed electrospun fibers of SMA and polyvinyl alcohol (PVA) for wound healing. SMA was a shell of the fibers containing different concentrations of silver nanoparticles (AgNPs) as antibacterial agents and a core of PVA containing allantoin as a healing agent. The results indicated that SMA allowed an immediate but controlled release of the antibacterial agent (Tamayo et al. 2022).

The development of nanogels marked a significant advancement in medication delivery (Le et al. 2022). This allowed for gel–cell contact and systemic delivery. Nanogels can have several structures. This may include core–shell nanoparticles, hollow nanogels, double-walled, and yolk shell structures (Ahmed et al. 2020). Nanogels possess the ability to encapsulate a significant amount of drugs due to their unique swelling properties. Not only do they have a higher drug loading capacity than other nanoparticles, but they also release the entrapped molecules at a sustained rate (Shah et al. 2020). This makes nanogels an attractive option for drug delivery systems (Hassan et al. 2023; Suhail et al. 2023).

Dementia from Alzheimer's disease (AD) is the most commonly diagnosed neurodegenerative disease. As a result of the disease, aggregation of beta-amyloid, abnormal protein (Aβ), deposits in the brain (Singh et al.), cholinergic neurons in the brain are lost, and the level of acetylcholine (ACh) in the brain is reduced by acetylcholinesterase (AChE) enzyme. Several attempts have been afforded to prevent the effect of (Aβ) and (AChE) enzyme activities (Srivastava et al. 2019; Tripathi et al. 2019, 2024; RamaKrishna et al. 2024). AChE inhibitors or anti-cholinesterase agents increase the level and duration of the neurotransmitter action, as they prevent the cholinesterase enzyme from degrading ACh. AChE inhibitors are classified as either irreversible or reversible based on their manner of action. Generally, an irreversible inhibitor inactivates an enzyme by bonding covalently to a particular group at the active site. A reversible inhibitor inactivates an enzyme through noncovalent, reversible interactions. Reversible inhibitors, whether competitive or non-competitive, generally have therapeutic applications, while irreversible AChE activity modulators are often associated with toxic effects (Colovic et al. 2013).

Donepezil is a second generation of reversible AChE inhibitor licensed by the US FDA for the treatment of mild-to-severe Alzheimer's disease (AD). In addition, Donepezil has the ability to delay the deposition of Aβ. Several clinical studies state that Donepezil improves cognitive function in patients receiving the higher dose. However, the higher drug dose increased occurrence of cholinergic side effects that may include anorexia, abdominal pain, cardiac vagal tone, and gastrointestinal anomalies—nausea. This pharmacological molecules with poor water solubility (0.0045 mg/mL) require formulation with micellar or nanoparticles (NPs) for increased bioavailability and avoided its side effect (Piazza et al. 2014; Dhas and Mehta 2020).

Due to their biocompatibility and biodegradability, polymeric nanoparticles are suitable for control and sustained delivery systems. Although NPs can increase brain medication delivery through numerous mechanisms, particles larger than 250–300 nm are ineffective due to limited penetration into intracellular and paracellular areas (Bourquin et al. 2018; Singh et al. 2019). Considerable attention has been paid to NPs based on CS as promising candidates for transport therapeutics through BBB (Caprifico et al. 2020; Cortés et al. 2020). One of the unique features of CSNPs is their ability to overcome biological barriers (Herdiana et al. 2022). Hence, CSNPs form ionic connections with endothelial cells that ease drug overpass the BBB through adsorptive transcytosis (Caprifico et al. 2020). Polymeric nanoparticles made from low-molecular-weight chitosan are promising for non-invasive therapeutic transport across the blood–brain barrier, offering advantages, such as low toxicity, biodegradability, and mucoadhesivity.

In this work, nanoparticles of CS-PEC with SMA derivative were prepared for Alzheimer’s disease treatment. The aim is to introduce a new strategy for drug delivering through the blood–brain barrier (BBB). This barrier remains a challenge, because only small lipophilic molecules and the less than 400 Daltons molecules can diffuse through it (Bourquin et al. 2018). In this regard, low-molecular-weight sulfonated SMA (S-SMA) was prepared. PEC was formed by mixing low-molecular-weight polycation and polyanion β-CS and S-SMA, respectively. The investigation was carried out with different ratios of S-SMA. Donepezil was encapsulated in the optimum ratio and in vitro drug release was studied. Additionally, acetyl cholinesterase (AChE) inhibitory assay was investigated.

## Materials and methods

### Materials

**Scheme 1:**
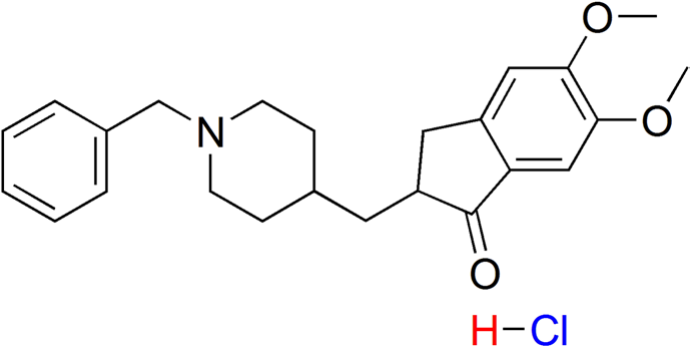
Donepezil hydrochloride (DH)

Chitosan, poly-(1, 4-β-D-glucopyranosamine), > 90%, molecular weight 10-20KD was purchased from Bio Basic Inc., Canada. Styrene was a product of Aldrich. Maleic anhydride (99%) was re-crystallized from chloroform prior to use, benzoyl peroxide (BPO), was re-crystallized from methanol, and chlorosulfonic acid were Merck products. Donepezil hydrochloride (DH) **(**Scheme 1) obtained from Acros. Acetone, acetic acid, ethylene chloride, methanol, and toluene were used as received.

### Synthesis of low-molecular-weight styrene–alt–maleic anhydride (SMA)

**Scheme 2:**
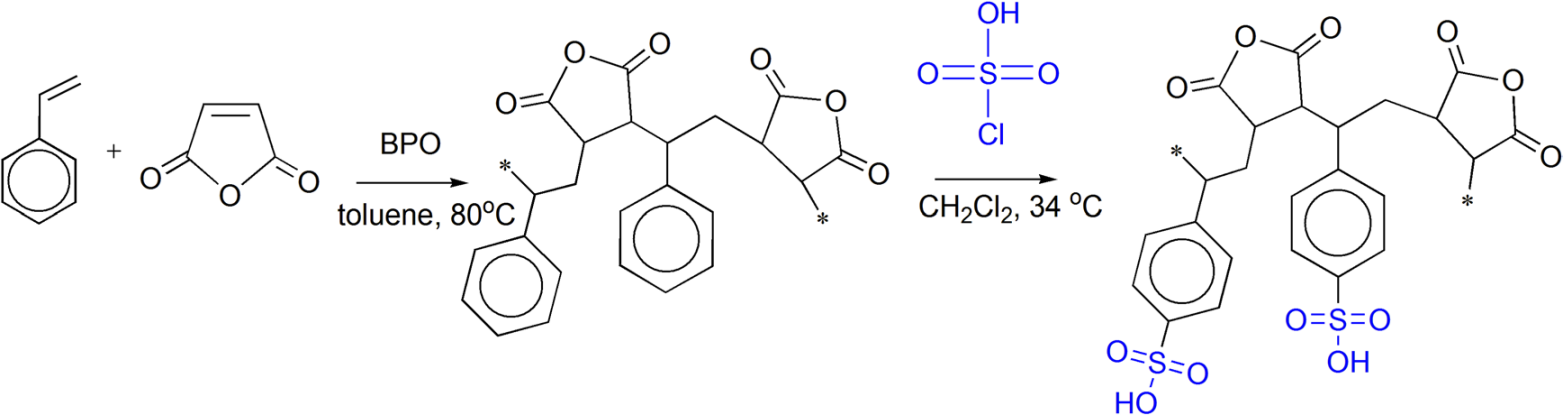
Synthesis and sulfonation of styrene–alt–maleic anhydride

SMA was prepared by the free-radical polymerization according to previous reference (Lai et al. 2008). In a 500 ml four-necked flask, 9.8 g of re-crystallized maleic anhydride, 10.4 g of destabilized styrene, and 0.8 mol of BPO were stirred with 200 ml of distilled toluene at room temperature until a clear solution was obtained (Scheme 2). Then, the reaction mixture was heated to 80 °C and continuously stirred under inlet of nitrogen. After 3 h, the copolymer gradually precipitated. The filtration of the white precipitate was carried out after cooling. The copolymer (SMA) was dried to constant weight at 70 °C. Finally, SMA was re-precipitated from acetone on methanol.

#### Determination of SMA molecular weight

Using Ostwald viscometer, the viscosity of different concentrations of SMA solution in acetone was measured. The reduced viscosity for each solution (η_red_) was determined. Then, the intrinsic viscosity [η] was derived from the plot of η_red_ against different concentration. Mark–Houwink molecular weight (Ravindra et al. 1998) was implemented for the calculation of the molecular weight

1 $$\left[\eta \right]=k{M}^{a}$$

The parameters, k and a, are dependent on the polymer–solvent system. For SMA solutions in acetone, k equals 8.96 × 10^–5^ mL/g and an equals 0.74 (Akar and Rahimian 2001).

### Synthesis of sulfonated (styrene–alt–maleic anhydride) (S-SMA)

0.37 mol of SMA were suspended in 480 mL ethylene dichloride. Then, 0.28 mol of chlorosulfonic acid dissolved in 50 mL of methylene dichloride was then gradually added with no required cooling or heating (Vachon et al. 2013). After the chlorosulfonic acid complete addition, the temperature remained for one and half hour at about 34 °C. The product, a light tan solid, was removed by filtration and air-dried.

### Preparation of sulfonated styrene–maleic anhydride/chitosan polyelectrolyte complexes nanogel

A certain amount of S-SMA at pH = 5.5, as depicted in Table 1**,** was added drop wise to 20 ml of 0.5% β-CS solution with vigorous mixing by mechanical stirring (6000 rpm). The formation of polyelectrolyte complex was observed by solution turbidity occurrence. By centrifugation of the solution at 3000 rpm for 20 min, the nanoparticles were collected. Then, the product was washed with water and acetone. The nanogel was dried using vacuum freeze dryer.

**Table 1:** The nanogel composition and zeta potential

Sample code	β-CS (%)	SSMA (%)	ζ potential (mV)
CS	0.5	––	39.6
CS-S1	0.5	0.1	35.7
CS-S2	0.5	0.2	32.6
CS-S3	0.5	0.3	28.7

### Preparation of cholinesterase inhibitor nanoparticles

Donepezil hydrochloride 6 mg were added to 100 ml of 0.5% β-CS solution in 1%acetic acid and stirred for 5 min. Then, a certain amount of 0.3% sulfonated SMA was added drop wise with vigorous stirring (6000 rpm). After 30 min, the suspension was subjected to centrifugation for 20 min at 3000rpm.

### Donepezil-loaded nanoparticles encapsulation efficiency and loading capacity

After separation of Donepezil-loaded nanoparticles from suspension by centrifugation, the supernatant was collected and drug content (free drug) in the supernatant was determined by the UV spectrophotometric method at 268 nm.

The Donepezil encapsulation efficiency (EE) and drug loading (DL) were calculated according to the following equations:

2 $$\%EE=\left(\frac{A-B}{A}\right)\times 100$$

3 $$\%DL=\left(\frac{A-B}{C}\right)\times 100$$

where A is the total amount of Donepezil, B is the free amount of Donepezil, and C is the weight of nanoparticles.

### In vitro* release studies*

The in vitro drug release study was performed using the dialysis bag method. A certain weight of nanoparticles equivalent to 200 μg of the drug was placed in a cellulose dialysis bag, (MWCO 12,000 g/mole; Sigma, St. Louis, USA) and both ends were sealed.Then, under sink conditions, the dialysis bags were immersed in phosphate buffer solution (PBS) (pH 7.4) using a water bath shaker at 37 ± 0.5 °C at 70 rpm. Samples were withdrawn at regular time intervals and the same volume was replaced with fresh PBS. Each sample was measured by UV spectrophotometer at wavelength 271 nm. The measurements were performed in triplicate and averaged.

### Mechanism of drug release

A variety of kinetic equations were investigated to the in vitro release studies of drug-loaded nanoparticles, including zero, and first, orders, Higuchi, Hixson–Crowell, and Korsmeyer–Peppas models. These theoretical models describing drug release from polymeric systems. Higuchi model describes drug release as a diffusion process based on Fick’s law according to the following equation:

4 $$\frac{{M}_{t}}{{M}_{\infty }}= K{t}^{0.5}$$

where M_t_ and M_∞_ are donated for cumulative amounts of released drug at time t and in finite time, respectively. K is the Higuchi constant (Hemmati and Ghaemy 2016). The Korsmeyer–Peppas model considering the deviation from Fick’s law that may lead to anomalous behavior (Zhu et al. 2023). This model is described by the following equation:

5 $$\frac{{M}_{t}}{{M}_{\infty }}= K{t}^{n}$$

where K is the kinetic constant and n is an exponent, characterize different release mechanisms.

### Cytotoxicity assessment

Cytotoxicity studies were completed on human normal cell line (HFB4). These cells were cultured in a saturating humidity and in full-growth DMEM medium supplemented with 10% FBS, L-glutamine (2 mM), streptomycin (100 μg/mL), and penicillin (100 IU/mL) in the CO_2_ incubator at 37 °C, 5% CO_2_. The cells were seeded at concentration 5000 cells per well into 96-well tissue culture plate and incubated overnight under conditions described above. Samples were sterilized under ultraviolet (UV) light for 30 min in a laminar flow before extraction, while the extracted media were filter-sterilized using a 0.22 μm syringe filter. According to ISO 10993–12, extraction of 0.1 g of samples to 1 ml of cell growth medium at 37 °C were prepared after 24 h. Afterward, the extracts were added to the cells in quadruplicates (*n* = 4) and incubated for 24 h. The amount of living cells was determined by the MTT assay as described by previous reports (Nada et al. 2016, 2018) (Ragab et al. 2019).

### Acetyl cholinesterase (AChE) inhibitory activity

The acetylcholinesterase (AChE) activity was assayed according to a reported spectrophotometric method (Khan et al. 2010).

Different samples were suspended in DMSO (HPLC). The reaction mixture contained 150 μL of (100 mM) sodium phosphate buffer (pH 8.0), 10 μL of 5,5-dithiobis-(2-nitrobenzoic acid) (DTNB, Sigma), 10 μL of test compounds solution, and 20 μL of acetyl cholinesterase solution (Electric-eel AChE-Sigma) were mixed and incubated for 15 min (37 °C). Then, it was followed by the addition of 10 ml of acetylthiocholine (Sigma) to initiate the reaction. Hydrolysis of acetylthiocholine was monitored by the formation of yellow 5-thio-2-nitrobenzoate anion measured by UV–Vis Shimadzu spectrophotometer-USA at a wavelength of 412 nm (15 min). All reactions were performed in triplicate in 96-well micro-plate.

The inhibitory activity was expressed as % inhibition and was calculated as follows:

6 $$\%I=\frac{{A}_{c}- {A}_{s}}{{A}_{c}}\times 100$$

where Ac: absorbance of the control and As: absorbance of the sample.

### Statistical analysis

One-way ANOVA was used for comparing groups followed by Tukey test for multiple comparisons using Graphpad Prism software, version 5 (Inc., USA). The difference was considered significant when p < 0.05, where a: significantly different from positive control (DH), b: significantly different from CS group, and c: no significant difference.

### Characterization

#### FTIR spectroscopy

FTIR spectra were measured by spectrometer, Bruker Vector 22, Germany, with resolution of 4 cm^−1^. The range of measurements was 400–4000 cm^−1^.

#### Dynamic light scattering (DLS)

Zetasizer, Nano-S, produced by Malvern, was the instrument used for measuring the particles size and zeta (ζ) potential at 25 °C in triplicate considering the refractive index of 1.52.

#### Thermal analysis

Thermogravimetric TGA analysis was accomplished at a heating rate of 10 °C/min under nitrogen atmosphere by Shimadzu TGA-50H.

#### XRD analysis

Philip’s X-ray diffractometer PW1390 was used to perform wide-angle X-ray diffraction (WAXRD) measurements, using Ni-filtered CuKα radiation with a wavelength of 1.5404 Å at a generator voltage of 40 kV and tube current of 30 A.

#### Transmission electron microscopy (TEM)

The nanoparticles were placed on a carbon-coated grid left for a min to allow the nanoparticles to adhere to the carbon substrate. The nanoparticles were stained with aqueous phosphotungstic acid (PTA) solution, 1% (w %) maintained for 60 s, and then air-dried. Then, they were analyzed using a JEOL-JEM2100 (USA) high-resolution transmission electron microscope (TEM) at 200 kV.

#### The UV–visible spectroscopy

The UV–visible spectra of the samples were measured using Agintal UV–visible spectrophotometer (USA).

## Results and discussion

### Preparation of low-molecular-weight styrene–alt–maleic anhydride (SMA)

The intrinsic viscosity [η] of SMA was estimated from the viscosity measurements using Ostwald viscometer. It was found to be equal 1.1909 dL/g. According the Mark–Houwink equation, *M*_*v*_ was estimated the following equation parameters for SMA in acetone (Akar and Rahimian 2001):

$$\eta =8.69\times {10}^{-5}{M}_{v}^{0.74}.$$

The viscosity-average molecular weight was found to be equal *M*_*v*_ = 10^5.5^ g/mol.

### Synthesis of sulfonated (styrene–alt–maleic anhydride) (SSMA)

The spectra of SMA and SSMA are shown in Figure 2. The peaks at 1844.1 and 1780 cm^−1^ implied the presence of anhydride groups resulting from the carbonyl absorption in the five-membered rings (Ali et al. 2018). The strong characteristic peak at 1176.22 cm^−1^ that is assigned to the stretching S = O confirms introduction of the sulfonic acid groups. The characteristic absorption peaks at 1176. 22 and 1017.84 cm^−1^ correspond to the asymmetric S = O and symmetric O = S = O stretching vibrations of the sulfonate groups, respectively (Roeges and Baas 1994) (Jiang et al. 2024). The two peaks at 705.35 and 691.08 cm^−1^ are considered relate to the C–S and S–OH-stretching vibration, which were reported (Heravi et al. 2014) to lie between 600 and 800 cm^−1^ and 600 and 700 cm^−1^, respectively.

### Preparation of sulfonated styrene–maleic anhydride/chitosan polyelectrolyte complex nanogel

**Fig. 1:**
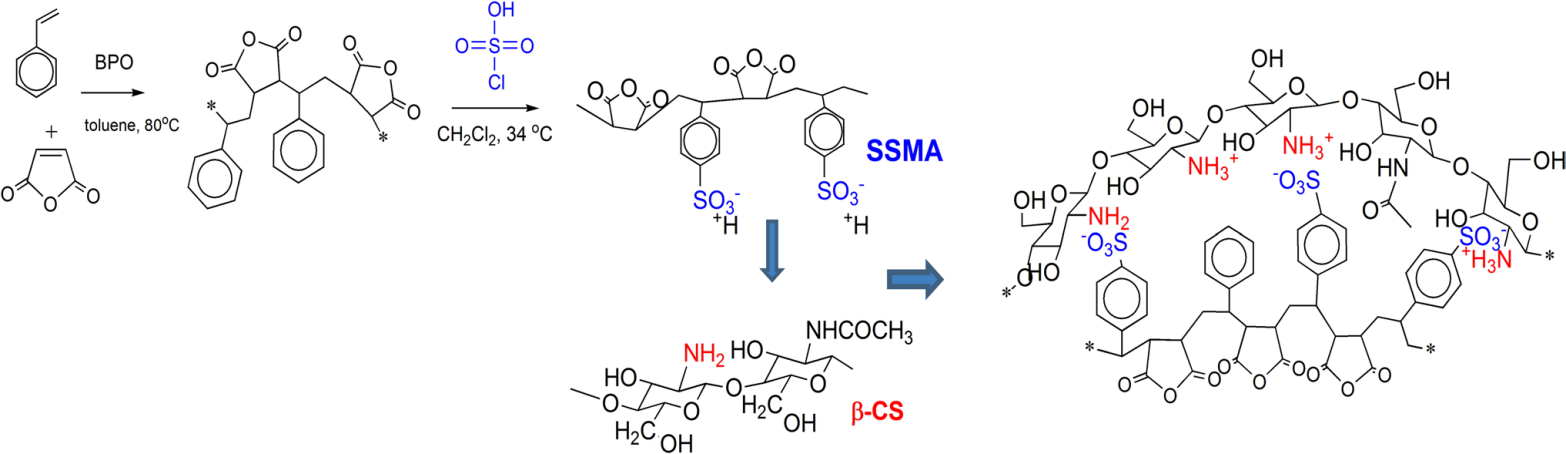
The proposed structure of β-chitosan PEC

Developing PECs is considered a green and energy efficient process. Hence, PECs are developed in water by mixing oppositely charged polyelectrolyte solutions without the need of organic solvents or chemical cross-linkers in ambient temperature (Wu et al. 2020). The PEC based on polysaccharides, in particular CS, is of great interest in the pharmaceutical field because of their similarities to natural systems. The amino groups NH_2_ are the key functional groups for its interaction with other polyelectrolytes. Mainly, chitosan has two forms, named α- and β-chitosan. The α-CS possesses intra-chain, intra-sheet, and inter-sheet hydrogen bonds from the antiparallel sheets, whereas β-chitosan has no hydrogen bonds between two inter-sheets due to their parallel directions. Therefore, β-chitosan has a less crystallinity which lead to a better solubility than α-CS (Jung and Zhao 2012) and interaction with the oppositely charged polyelectrolyte (Fig. 1).

**Fig. 2:**
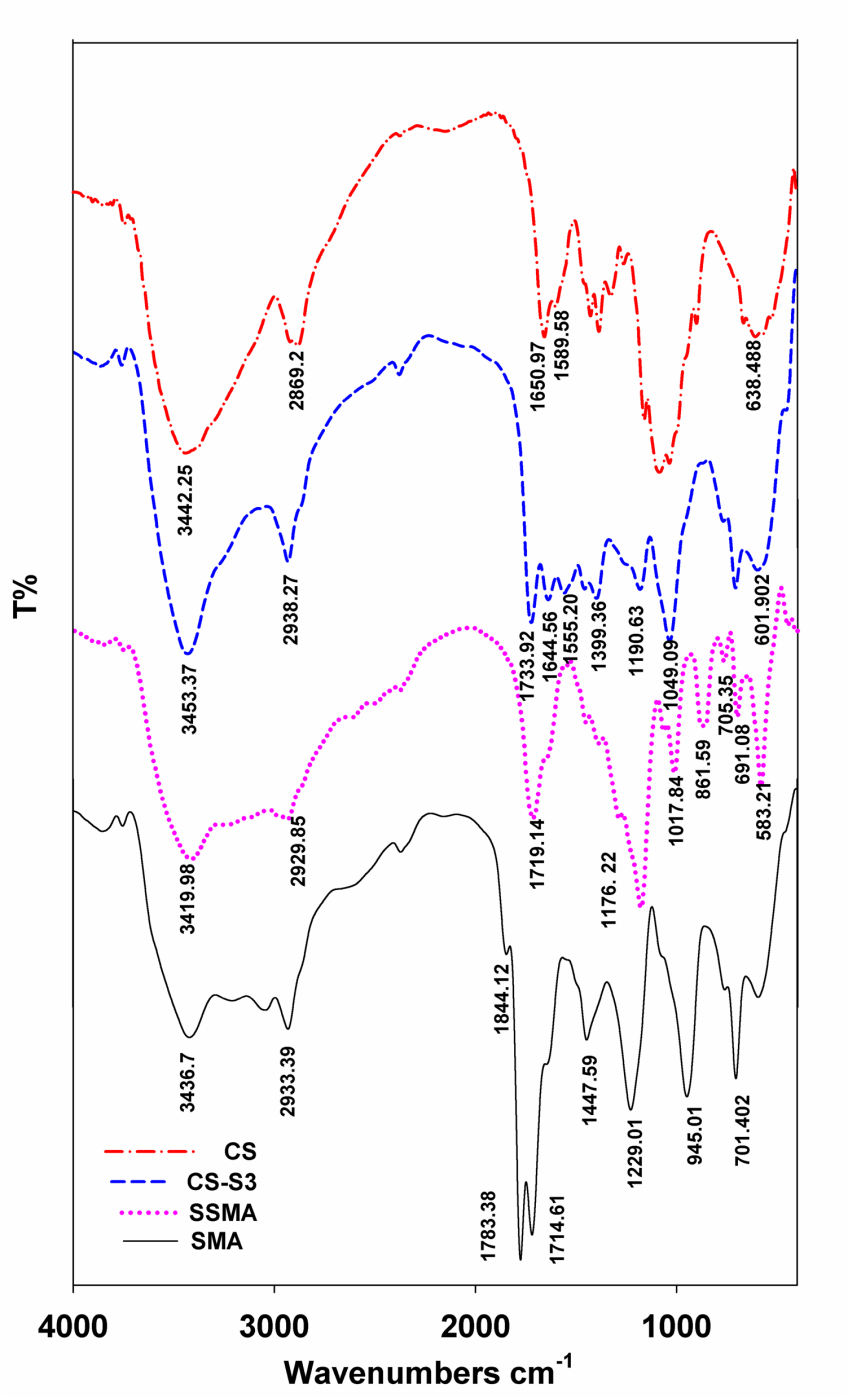
FTIR spectrum of CS, SMA, SSMA, and CS-S3

Figure 2 shows the IR-spectra of SSMA and β-chitosan and the obtained complex. CS’s characteristic peaks are existing at 3442 cm^−1^ (NH_2_ absorption band)_,_ 1589 cm^−1^ (N–H bending peak of amine I), and 1650 cm^−1^ (the amide II carbonyl stretching) (Ali et al. 2023).

The IR spectrum of the prepared complex CS-S3 revealed new peaks in the range of 1577–1507 cm^–1^ donated to symmetrical deformational vibrations of chitosan NH_3_^+^ groups. In addition, it illustrates the characteristic band for S-SMA in the region of 1719–1733 cm^−1^ (Roeges and Baas 1994). The presence of these peaks is clear evidence of the contribution of β-CS and S-SMA to the final polyelectrolyte complex composition. In addition, the decline of the broad band within 3000–3500 cm^−1^ of β-CS is attributed to the interaction of β-CS with the negatively charge sulfonate groups of SSMA.

### Particle size and zeta (ζ) potential analysis

**Fig. 3:**
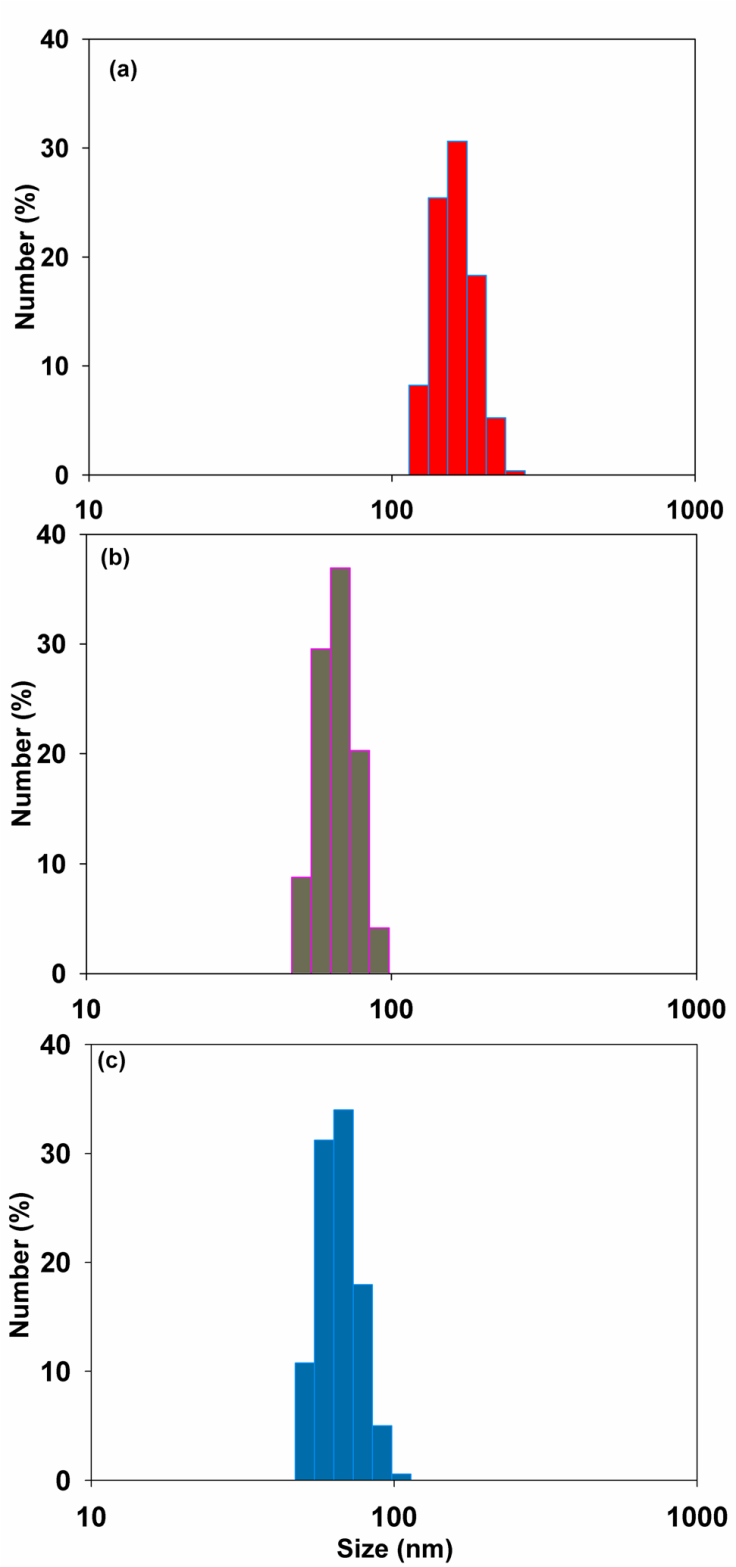
Effect of SSMA in feed ratio on the particle size of (**a**) CS-S1, (**b**) CS-S2, and (c) CS-S3

The average particle diameters of CS-SSMA polyelectrolyte complexes were determined by utilizing dynamic light scattering investigation. Figure 3 illustrates the average particle diameters of CS-SSMA polyelectrolyte complexes. The results revealed a decrease of the particle size with increasing the ratio of SSMA in the PEC. The average particle size decreases from 186 ± 0.8 for CS-S1 to 71 ± 0.03 for CS-S2. However, there is no drastic decrease when SSMA ratio increased to the value equals 0.3%. As shown in Fig. 3, the particle-size range was between 50 to 91 ± 0.6 nm for CS-S2, whereas for CS-S3, the range extends to be within 50 to 105 ± 0.5 nm. However, Fig. 3** (c)** shows that the frequency of smaller particle size is higher for CS-S3 compared to CS-S2. Therefore, CS-S3 was chosen for further investigations.

Often, ζ-potential analysis is the way by which double-layer properties could be described. Table 1 reveals the ζ-potential of CS complexes. Due to the cross-linking with electrostatic attraction, the ζ-potential of CS complexes decreases with increasing SSMA ratio. The zeta potential declined from 39.6 mV for CS to the value 28.7 mV for CS-S3.

### XRD analysis

**Fig. 4:**
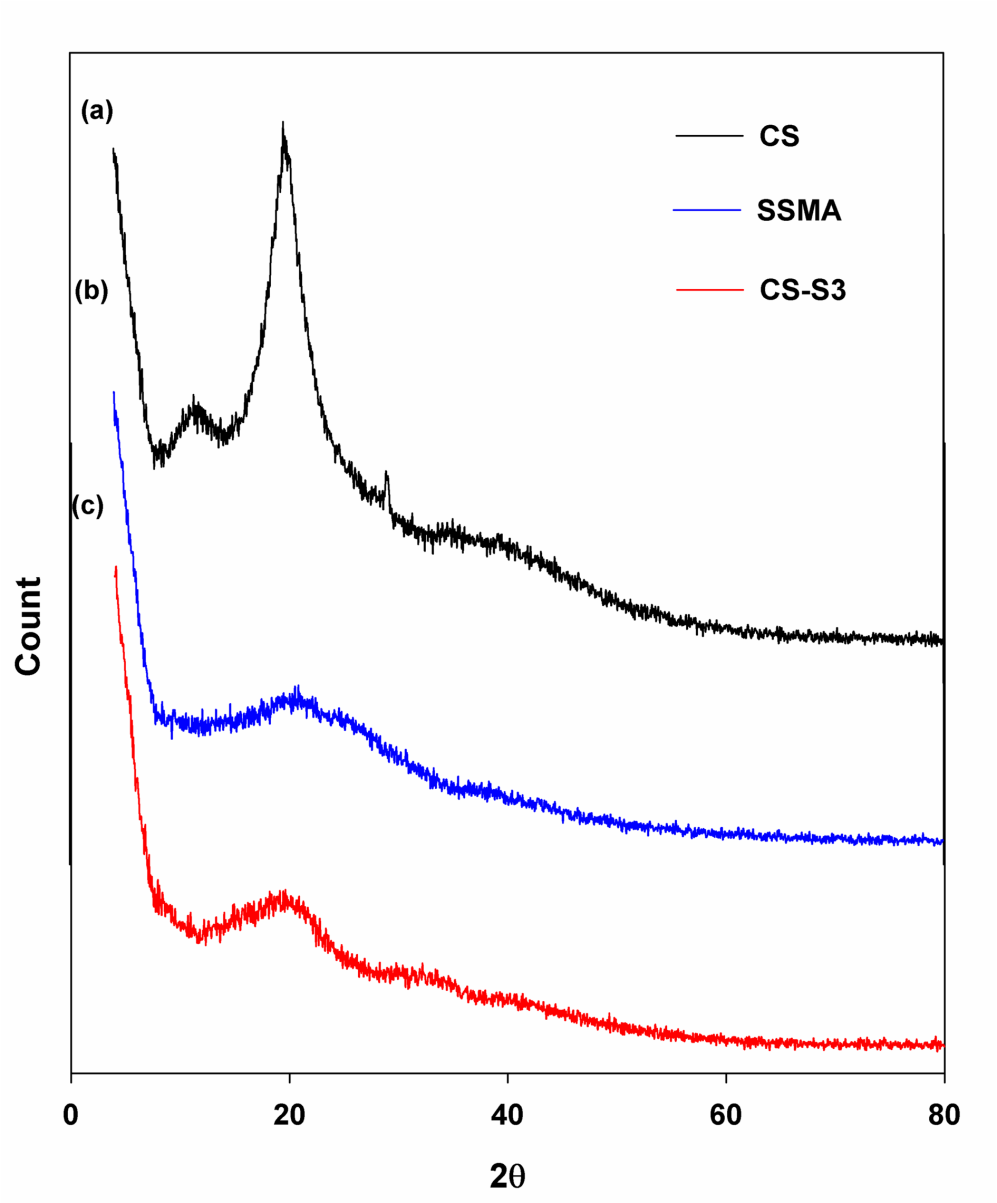
XRD analyses of (**a**) CS, (**b**) SSMA, and (**c**) CS-S3

Figure 4 shows the X-ray powder diffraction of CS, SSMA and CS-S3. The pattern of β-CS most likely exhibited two peaks around 19.66° and 28.9° which were indexed to the (020) and (110) planes of the crystalline lattice. Also, due to the absence of hydrogen bonds between two inter-sheets of its parallel arrangement, there is no crystalline peak at 2θ = 10^o^ (Jampafuang et al. 2019). In the case of CS-S3, the diffraction peak of CS at 19.6 °C is shifted to 15.7°, whereas the other peak at 28.9° is shifted to 31°and their intensities are weakened.

### Thermogravimetric analysis

**Fig. 5:**
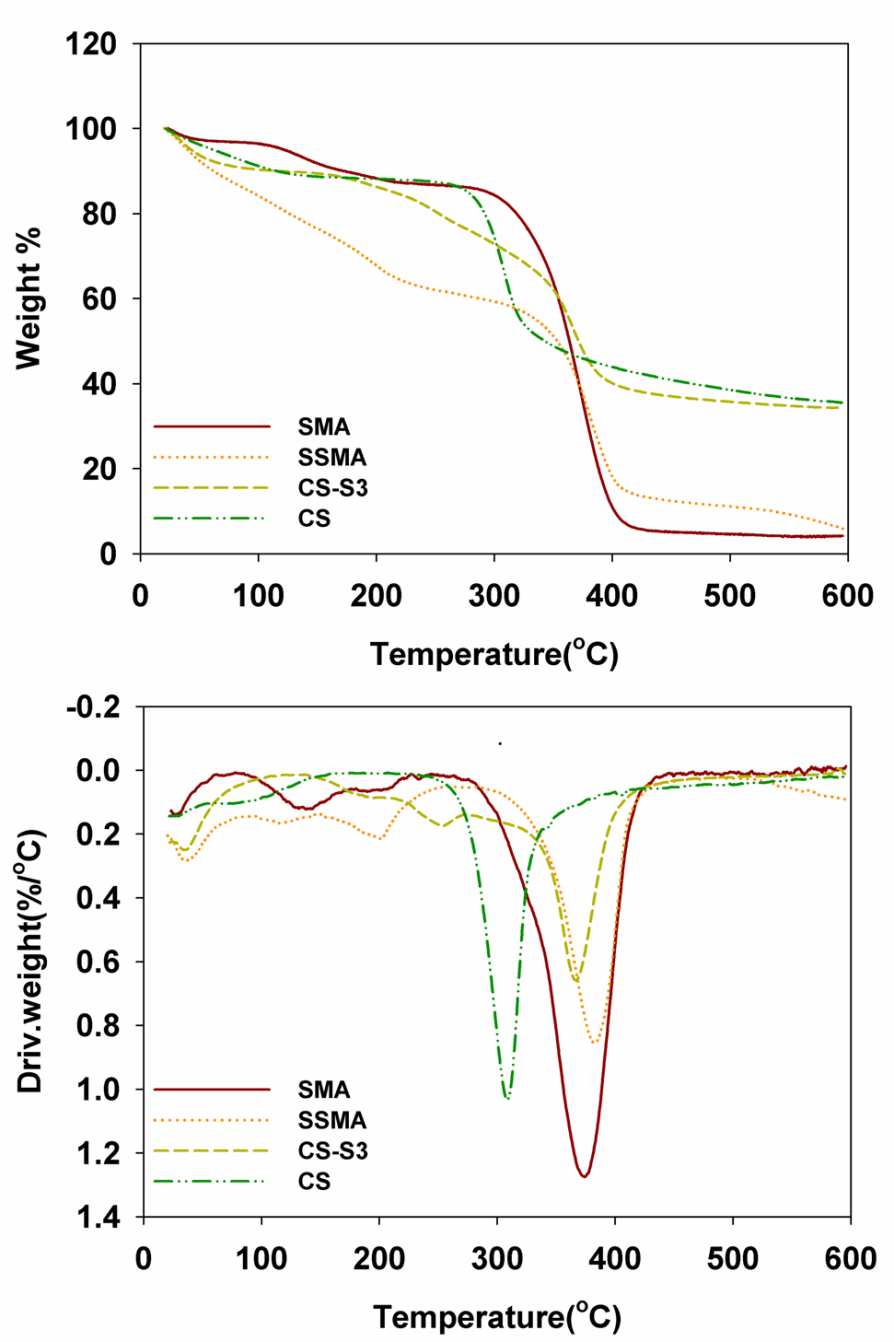
Thermogravimetric analyses of CS, SMA, SSMA, and CS-S3

Figure 5 illustrates the TGA of SMA, sulfonated SMA (SSMA), chitosan (CS), and polyelectrolyte complexes of chitosan and SMA (CS-S3). It is evident that the thermal stability of the polymers varies, with CS being the least stable and SMA being the most stable. Three weight loss stages can be clearly shown for (SSMA). The first occurs at 35–100 °C which is attributed to the loss of water as SSMA has a hygroscopic nature. The second occurs in the range of 150–225 °C the weight loss associated with the elimination of the sulfonic groups. The last loss stage at 350–500 °C is the final decomposition of SSMA. A summary of the TGA data is compiled in Table 2. Admittedly, it is not only the strong electrostatic interaction between CS and SSMA afforded the high thermal stability of CS-S3 but also the presence of hydrogen bonding and the hydrophobic interaction.

**Table 2:** Thermogravimetric analysis data of the samples

Sample	T_initial_	T_final_	T_max_	Wt loss%	Remaining wt.% at 600 °C
SMA	279.00	400.00	374.00	96.00	4.13
SSMA	198.00	596.00	382.00	91.70	8.13
CS-S3	216.00	596.00	367.00	65.70	34.30
CS	273.00	331.00	308.00	64.40	35.60

### Transmission electron microscopy (TEM) analysis

**Fig. 6:**
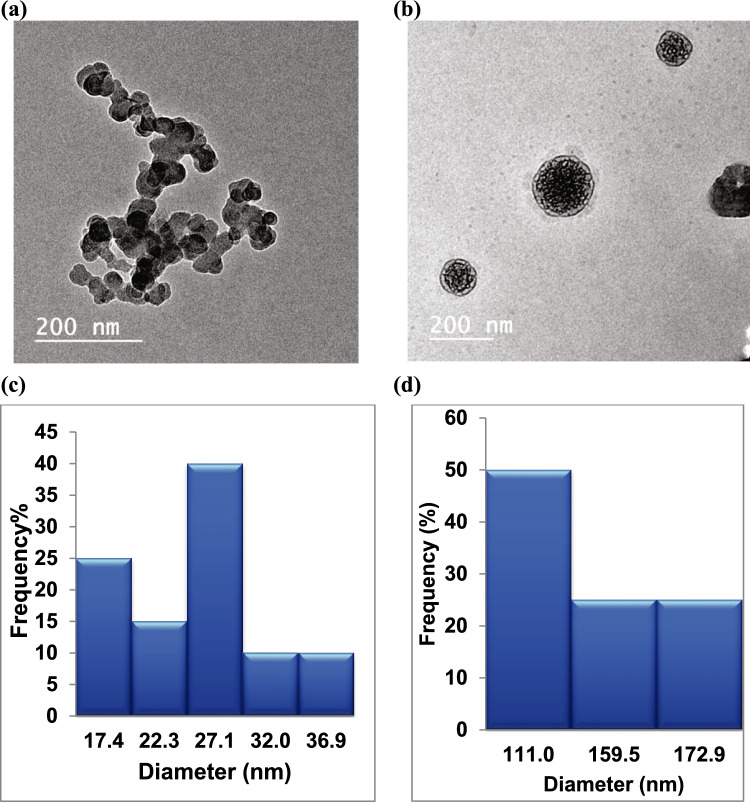
TEM images (**a**) and (**b**) and particle-size distribution (**c**) and (**d**) of CS-S3 and CS-S3-DH nanoparticles, respectively

Transmission electron microscopy (TEM) was used to determine the particle size of the optimum prepared nanoparticles**.** This technique is extremely useful as it enables a direct image for analyzing particle size (Online et al. 2017). Figure 6**a and b** illustrates TEM images of CS-S3 and CS-S3-DH nanoparticles, respectively. Rounded particles in the nanosize were observed. The measurement showed that their size was smaller than the size detected by DLS due to the swelling behavior of the nanogel. Polyelectrolyte complex cross-linking was recognized by the formation of a thin layer around the nanoparticles (Fig. 6**a and b)**. Typically, the size of CS-S3-DH nanoparticles is larger than CS-S3 as a result of encapsulating DH (Fig. 6**c, d**).

### Encapsulation efficiency (EE)

**Fig. 7:**
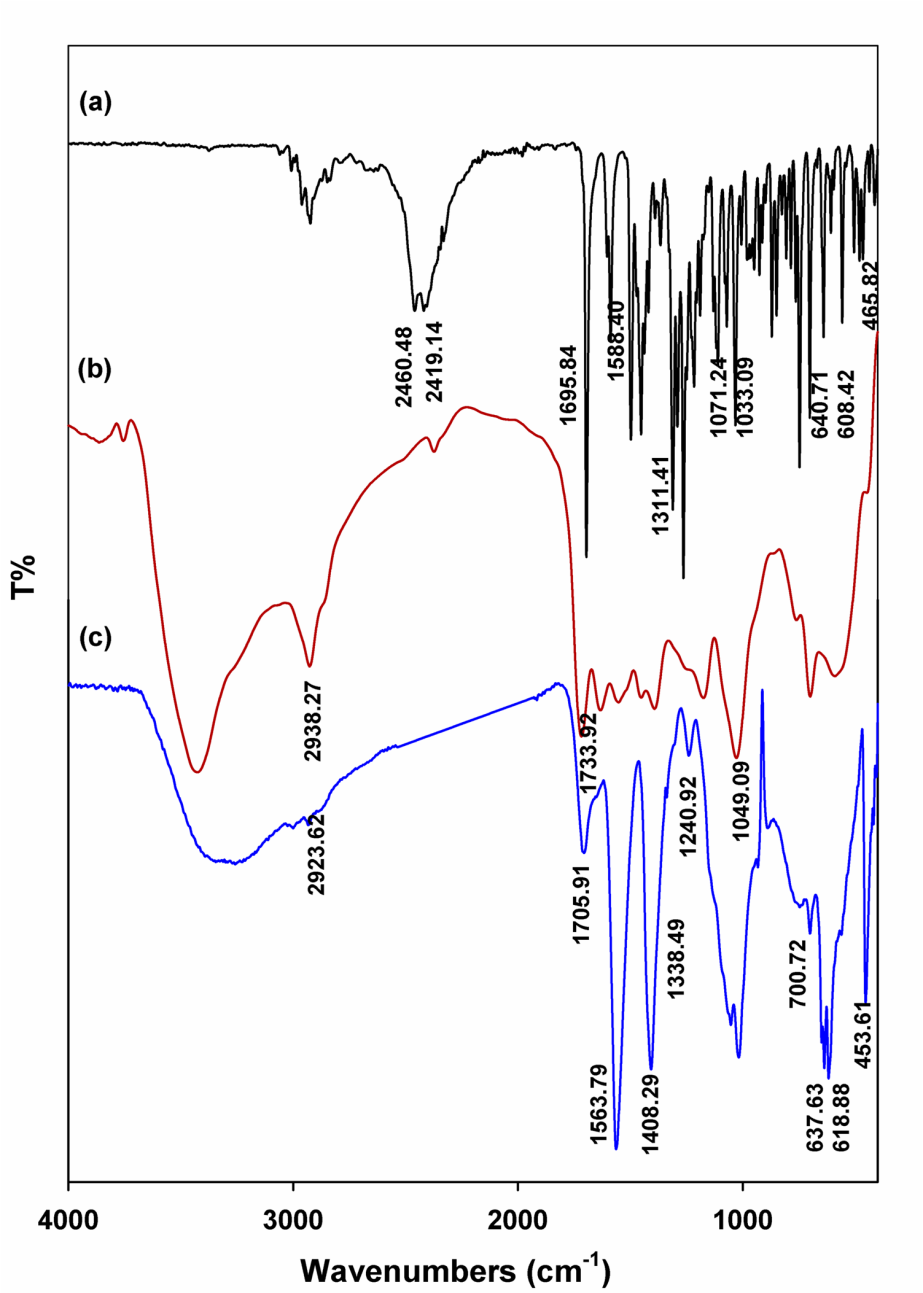
FTIR of (**a**) DH, (**b**) CS-S3, and (**c**) CS-S3-DH

The encapsulation (EE) of Donepezil hydrochloride (DH) was determined using UV–V is spectrophotometer at wavelength λ = 268 nm. The encapsulation (EE) was found to be 73% and the loading was 6.6%. The interaction of DH with CS-S3 was investigated by FTIR analysis. Figure 7 represents the FTIR spectra of DH, CS-S3, and CS-S3-DH. The encapsulation of DH in CS-S3 was confirmed by observing DH characteristic peaks in the CS-S3-DH spectrum (Fig. 7**c**). The C = O peak of pure DH (1695.84 cm^−1^) was observed at 1705 cm^−1^ and the intensity of this peak was declined in CS-S3-DH spectrum. Also, Fig. 7c shows other peaks characteristic of DH that appeared in the regions 1330–1240 cm^−1^ related to the C–N stretching of tertiary amine of DH. In addition, the distinctive peak of CS at 3382 cm^−1^ was widened because of hydrogen-bonding interaction between DH and CS-S3.

### In vitro* drug release from DHC-SSMA-CS NPs*

**Fig. 8:**
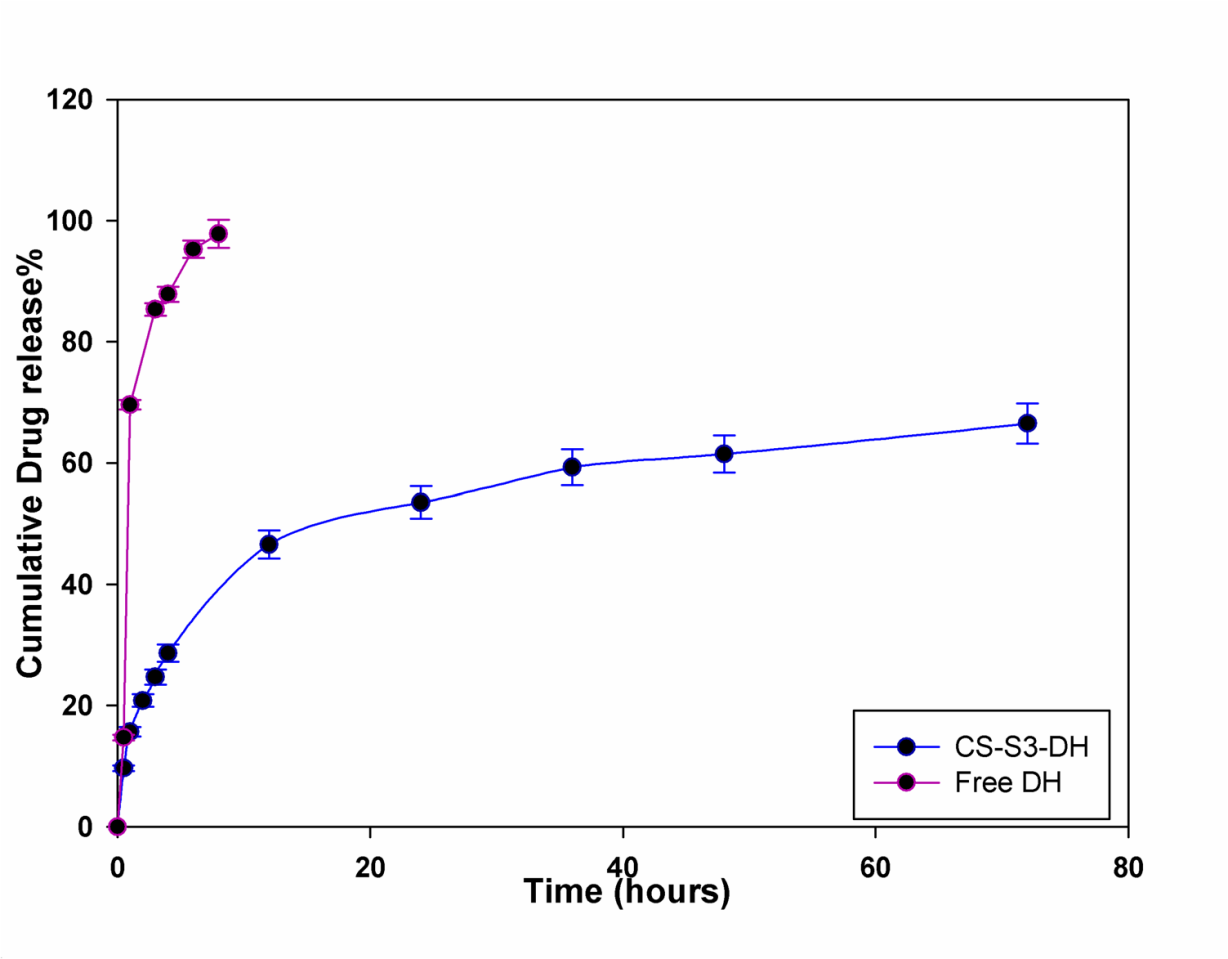
The in vitro release curve of DH from CS-S3 and free DH in phosphate-buffered saline (PBS) at 37 °C

The ability of a drug delivery system to extend the drug release in a controlled manner is of great interest for brain diseases due to the presence of BBB. Figure 8 represents the drug release profile of free DH and CS-S3-DH. It shows that while free DH is rapidly released within 8 h, CS-S3-DH exhibited a sustain release for 72 h. There are two stages in the drug release process of drug-loaded NPs. The initial stage with a rapid release (phase I) was observed in the range of 4 h. This stage was followed by a sustained long-term slow release (phase II). This behavior may be attributed to the loading behavior of the DH. Also, the presence of polymer conjugates with opposite charges stabilizes charge and control the release (Caprifico et al. 2020). The drug is likely to be adsorbed on SSMA-CS surfaces by a weak interaction that leads to the rapid release of the drug. While the slow diffusion of drug entrapped in the hydrophobic core of the particle leads to the slow release of the drug.

### In vitro* release kinetics*

The release kinetic parameters of CS-S3 were estimated using different mathematical models. Table 3 illustrates the coefficient (R^2^), K, and n from the non-linear regression. Compared to other studied models, the Higuchi kinetic model showed the highest R^2^ values for CS-S3. Therefore, the DH release from CS-S3 nanoparticle followed Fickian diffusion mechanism. For the determination of n, the exponent parameter of Korsmeyer–Peppas model, the data of the initial portion (i.e., Mt/M∞ < 60%) of the release curve was used. The value of n was 0.448 instead of 0.5 that may be considering an anomalous diffusion mechanism. However, in this case, Higuchi equation is the only model might be used in systems with a drug diffusion (Samaha et al. 2009). This is a good consistency with the fact that Higuchi model most likely represents drug release from matrix compromised water-soluble drugs.

**Table 3:** The release kinetics model parameters of DH from CS-S3

Model	Equation	Parameter	
		R^2^	K
Zero order	${M}_{t}={M}_{o}+{K}_{0}t$	0.8050	0.0324
		R^2^	K
First order	${lnM}_{t}=ln{M}_{o}+{K}_{0}t$	0.9248	−0.0003
		R^2^	K
Higuchi	$\frac{{M}_{t}}{{M}_{\infty }}= K{t}^{0.5}$	0.9958	1.7363
		R^2^	K
Hixson–Crowell	${M}_{o}^\frac{1}{3}-{M}_{t}^\frac{1}{3}=Kt$	0.9945	0.0416
		R^2^	n
Korsmeyer–Peppas	$\frac{{M}_{t}}{{M}_{\infty }}= K{t}^{n}$	0.9858	0.448

### In vitro* cytotoxicity assessment*

**Fig. 9:**
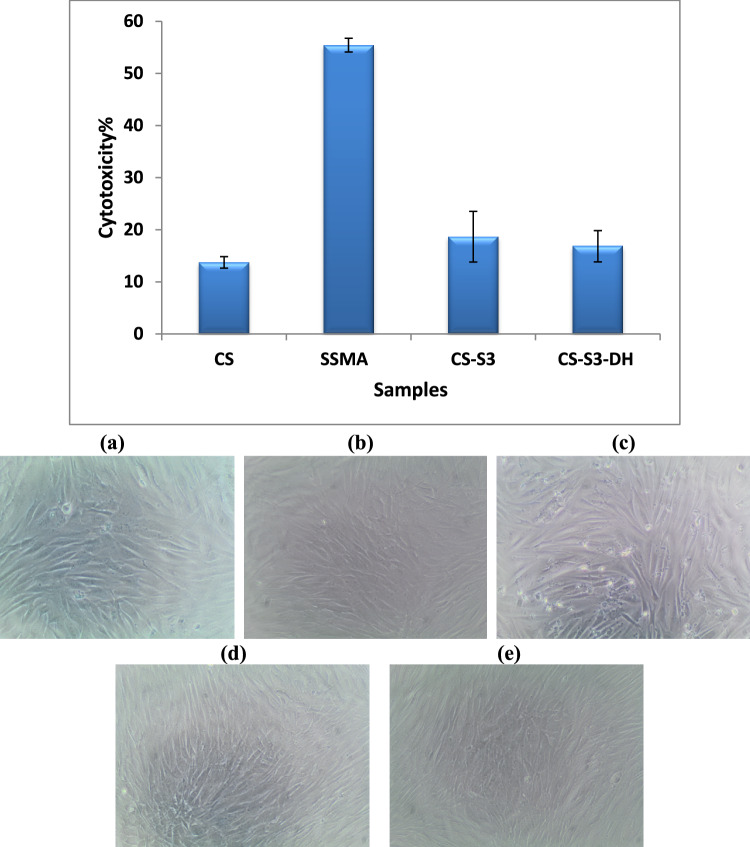
The cytotoxicity assessment at 100 μg/mL and cell morphology for (**a**) negative control (untreated), (**b**) β-CS, (**c**) SSMA, (**d**) CS-S3, and (**e**) CS-S3-DH

The cytotoxicity of the samples was investigated by 100 μg/mL using human normal cells, BJ1 fibroblast skin cells. The tested samples showed acceptable cytotoxicity except SSMA. Figure 9 shows that the highest cytotoxicity was observed to SSMA which attributed to acidity as a result of SO_3_ groups. However, when SSMA was combined with β-CS to form CS-S3 cell viability percentage increased to be 81.3%. The encapsulation DH had no significant effect on cell viability. The elongated spindle-like shape, normal cell morphology, was clearly observed for the negative control as well as the tested samples. The shape changed to a round shape when the cytotoxicity increased as in the case of SSMA as observed in Fig. 9c. By all, the data revealed that the prepared CS-S3 and CS-S3-DH nanoparticles are safe matrices for biological tissue.

### Acetyl cholinesterase (AChE) inhibitory activity

**Fig. 10:**
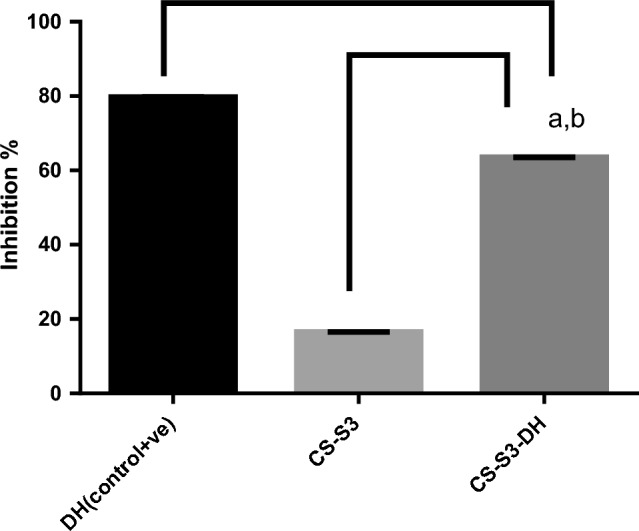
In vitro effects of different samples on acetylcholinesterase inhibition

Alzheimer's disease is linked to various obsessive traits, one of which is the deficiency of the neurotransmitter ACh. Therefore, the ongoing therapeutics is mainly based on increasing acetylcholine (ACh) levels to avoid the loss of memory. Acetylcholinesterase inhibitors (AChE) prevent the breakdown of acetylcholine (ACh) by inhibiting the enzyme that responsible of ACh deficiency. The AChE inhibitory activity was investigated for CS-S3 and CS-S3-DH, the encapsulated, nanoparticles. Figure 10 reveals that CS-S3 has AChE inhibitory activity of 16.5%, whereas CS-S3-DH 63.9%.

In vitro effects of different samples on acetylcholinesterase inhibition.

AChE inhibitory activity is expressed as percentage inhibition. Values are expressed as mean ± SD, n = 3 for each concentration. The letters “a” and “b” indicate: significant difference at P < 0.05 in comparison with positive control (DH);

## Discussion

Drug delivery materials can be manufactured using colloidal polyelectrolyte complexes (PECs) in a safe, efficient, and environmentally friendly manner. With no organic solvents, chemical cross-linker or surfactant, PECs are formed in water by mixing the oppositely charged polyelectrolyte solutions.

In the present study, cationic biopolymer CS was mixed with three different ratios of SSMA for the formation of the optimum nanosize PEC particles to incorporate a well-known reversible AChE, Donepezil drug.

The prepared PEC formulations were prepared by biocompatible polymers with low molecular weights (LMW) to generate suitable physicochemical characteristics dispersions (Hartig et al. 2007a; Choi et al. 2008). In addition, CS weight percentage was higher than SSMA to create a neutral core surrounded by excess CS in which provides the preferred nanosized and stabilized particles against aggregation (Schatz et al. 2004).

The average particle size decreases from 186 ± 0.8 for CS-S1 to 71 ± 0.03 for CS-S2 due to the formation of new bonds. Generally, there are multiple steps involved in PEC formation (Buriuli and Verma 2017c). In the first step, the chain configuration of polymers is drastically altered and a random primary complex is formed. After that, the secondary complex forms after the intra-complexes reorganize existing linkages. In this process, new bonds are formed, e.g., electrostatic bonds, hydrogen bonds, hydrophobic interactions, etc. (Hartig et al. 2007b). Finally, the formation of entangled aggregates, fibrils, ordered networks, or other stable structures is most likely occurred under certain conditions (probably due to hydrophobic interactions) (Wu et al. 2020). As shown in Fig. 3, the particle-size range was between 50 to 91 ± 0.6 nm for CS-S2, whereas for CS-S3, the range extends to be within 50 to 105 ± 0.5 nm. Figure 6**a and b** illustrates TEM images of CS-S3 and CS-S3-DH nanoparticles, respectively. Rounded particles in as previously reported (Ashrafi 2020), polyelectrolyte complex cross-linking was recognized by the formation of a thin layer around the nanoparticles in TEM images.

It was previously approved that a PEC system based on this LMW formulation has been found to be non-toxic, although their precursor polymers have demonstrated some toxicity (Hartig et al. 2007b). While SSMA polymer demonstrated some toxicity, it has been found that PEC based on this LMW polymer formulation is non-toxic.

The encapsulation (EE) of Donepezil hydrochloride (DH) was determined using spectral analysis; UV-V is spectrophotometer and FTIR. The sharp absorption band of C–N in DH structure appears at 1311.41 cm^−1^(Al-Sarayra et al. 2024). Characteristic bands of DH were observed in CS-S3-DH nanoparticles spectrum at 3585, 1705, 1583, 1407, and 1338 cm^−1^, related to O─H, C═O (stretch), C─N─C (stretch), and C─H (swing) bands, respectively (Guler et al. 2024).

DH is the salt of a basic drug with a pKa of 8.2. The salt is likely to be soluble at physiological pH (around 7.4), as the basic drug is partially ionized, and the conjugate acid (HA +) is relatively weak.

Table 4 illustrates the physical properties and release profile of CS-3S compared with nanoparticles based systems for Donepezil delivery. CS-3S-DH possesses the smallest size over other CSNPs. The release of DH from CS-3S-DH after 24 h was 53.4%. Then a sustained drug release pattern was observed over further hours (72 h). Based on the correlation coefficient (R^2^) values of both models, the Higuchi model appears to be the more suitable fitting model for both formulations. The Higuchi model is frequently utilized for calculating drug release from swellable polymer systems (Jeong et al. 2022).

**Table 4:** Nanoparticles-based systems as drug carrier for Donepezil

Nanoparticles’ composition	Particle size (nm)	EE %	DL %	Release %	Ref
Solid lipid	121.0	67.95	12.15	89.35% within 24 h	(Yasir et al. 2018)
Methoxy poly(ethyleneglycol)-polycaprolactone	107.15 ± 1.48		16.54 ± 1.21	40% within 1 h followed by a sustained release pattern till 96 h	(Krishna et al. 2019)
Chitosan/sodium tripolyphosphate	135 ± 6	70.4 ± 0.8	––-	67.8 ± 1.6% in 24 h	(Amjad and Alotaibi 2020)
Mannose-coated (poly D, L-lactide-*co*-glycolic acid) nanoparticles	179.4	82.52 ± 3.26	6.8 ± 1.01	released 80% of the drug within 48 h,	(Handa et al. 2023)
Solid lipid	87.2 ± 0.11	93.84 ± 0.01		70% after 24 h	(Topal et al. 2023)
Chitosan/sodium tripolyphosphate	180.2		24.72	90% drug release 12 h	(Garg et al. 2024)
Chitosan/SSMA	111 ± 0.4	73 ± 0.01	6.6	46% within 12 h followed by a sustained release pattern till 72	This work

## Conclusion

Polyelectrolyte complexes’ nanogel for DH delivery was successfully prepared from β-CS and synthesized sulfonated SMA. Spectral analysis confirmed the chemical composition of the prepared PECs. The results obtained from DLS revealed a sharp decrease of the average particle size from 154 ± 06 to 69 ± 0.6 with increasing the S-SMA from 0.1 to 0.3%. The in vitro study of DH release shows a sustain release for 72 h. The in vitro results of the AChE inhibitory activity showed that CS-S3-DH had 63.9% inhibitory activity. The finding showed a promising DH carrier for the treatment of Alzheimer's disease and encouraged for further investigation in animal model. 

## Data Availability

Data will be made available on request.
